# Novel Therapies for the Treatment of HER2-Positive Advanced Breast Cancer: A Canadian Perspective

**DOI:** 10.3390/curroncol29040222

**Published:** 2022-04-13

**Authors:** Cristiano Ferrario, Anna Christofides, Anil Abraham Joy, Kara Laing, Karen Gelmon, Christine Brezden-Masley

**Affiliations:** 1 Department of Oncology, McGill University, Montreal, QC H3A OG4, Canada; 2 Jewish General Hospital, Montreal, QC H3T 1E2, Canada; 3 IMPACT Medicom Inc., Toronto, ON M6S 3K2, Canada; anna@impactmedicom.com; 4 Department of Oncology, University of Alberta, Edmonton, AB T6G 2R3, Canada; ajoy@ualberta.ca; 5 Cross Cancer Institute, Edmonton, AB T6G 1Z2, Canada; 6 Faculty of Medicine, Cancer Care Program, Memorial University, St. John’s, NL A1B 3V6, Canada; kara.laing@easternhealth.ca; 7 Department of Medical Oncology, University of British Columbia, Vancouver, BC V6T 1Z4, Canada; kgelmon@bccancer.bc.ca; 8 BC Cancer, Vancouver, BC V5Z 1G1, Canada; 9 Department of Medicine, University of Toronto, Toronto, ON M5S 1A8, Canada; christine.brezden@sinaihealth.ca; 10 Mount Sinai Hospital, Toronto, ON M5G 1X5, Canada

**Keywords:** breast cancer, HER2, oncology, human epidermal growth factor receptor 2

## Abstract

The advent of anti-HER2 targeted therapies has dramatically improved the outcome of HER2-positive breast cancer; however, resistance to treatment in the metastatic setting remains a challenge, highlighting the need for novel therapies. The arrival of new treatment options and clinical trials examining the efficacy of novel agents may improve outcomes in the metastatic setting, including in patients with brain metastases. In the first-line setting, we can potentially cure a selected number of patients treated with pertuzumab + trastuzumab + taxane. In the second-line setting, clinical trials show that trastuzumab deruxtecan (T-DXd) is a highly effective option, resulting in a shift from trastuzumab emtansine (T-DM1) as the previous standard of care. Moreover, we now have data for patients with brain metastases to show that tucatinib + trastuzumab + capecitabine can improve survival in this higher-risk group and be an effective regimen for all patients in the third-line setting. Finally, we have a number of effective anti-HER2 therapies that can be used in subsequent lines of therapy to improve patient outcomes. This review paper discusses the current treatment options and presents a practical treatment sequencing algorithm in the context of the Canadian landscape.

## 1. Background

In Canada, access to new treatments is complex, dependent on both Health Canada approval and provincial funding. Once a file is submitted to Health Canada, it may take up to one year to obtain approval, depending on the type of regulatory submission filed [1]. Subsequently, oncology drugs undergo health technology assessment (HTA), either by the Institut National d’Excellence en Santé et en Services Sociaux (INESSS) in Quebec or by the Canadian Agency for Drugs and Technologies in Health (CADTH) for the rest of Canada [2,3,4]. These bodies provide information on the clinical and economic evidence about the drug and recommendations to advise provincial decision-makers on funding. The HTA process takes approximately 10 months and is followed by a review by the Pan-Canadian Pharmaceutical Alliance (pCPA), which negotiates pricing with pharmaceutical companies and provides recommendations to provincial health authorities, after which provinces can decide whether to fund the treatment [4,5]. This last step can take approximately 13 months, and additional time is required before the drug is listed provincially and available to patients. However, CADTH’s recommendations are not a guarantee of funding, as each drug program can decide whether to fund a drug in an individual province. To fill the gap between Health Canada approval and public funding, a time-limited patient support program is often made available by pharmaceutical companies.

Breast cancer is the second most commonly diagnosed cancer in Canada and the most frequently diagnosed in women, with the exception of non-melanoma skin cancer [6]. Although survival rates have improved in recent years, breast cancer remains the second most common cause of cancer deaths in women, representing an estimated 5400 deaths in 2021. The human epidermal growth factor receptor 2 (HER2) subtype accounts for 15% to 20% of breast cancers and is historically associated with a poor prognosis [7,8]. However, the advent of anti-HER2 targeted therapies has dramatically improved outcomes, particularly in early-stage disease [7,8]. Unfortunately, resistance to treatment in the metastatic setting remains a challenge, highlighting the need for novel therapies in this subgroup [9]. 

**Figure 1:**
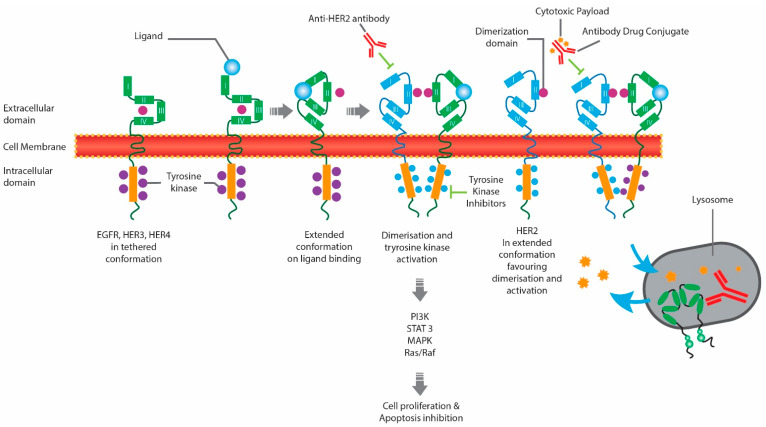
Mechanism of Action of Anti-HER2 Therapies. EGFR, epidermal growth factor receptor; HER2, human epidermal growth factor receptor 2; HER4, human epidermal growth factor receptor 4.

Anti-HER2 therapies approved by Health Canada include monoclonal antibodies (MAbs), such as trastuzumab [10] and pertuzumab [11]; small-molecule tyrosine kinase inhibitors (TKIs), such as tucatinib [12], lapatinib [13], and neratinib [14]; and antibody-drug conjugates (ADCs), such as ado-trastuzumab emtansine (T-DM1) [15] and trastuzumab deruxtecan (T-DXd) [16] (Table 1). The different classes of anti-HER2 therapies target different regions within the HER2 pathway, as described in Figure 1 [17]. MAbs bind to the extracellular domain of HER2, inhibiting HER2 directly and indirectly engaging the immune system via antibody-dependent cellular cytotoxicity (ADCC). Small-molecule TKIs bind to the intracellular TK domains of HER2 and other HER receptors, while ADCs include the monoclonal antibody trastuzumab, bound to a cytotoxic agent.

There are no Canadian consensus guidelines for the treatment of advanced HER2-positive breast cancer. Treatments are chosen based on international guidelines, clinical trial data, and publicly available and funded drugs, the latter being the prime decision-maker despite not always being in line with international guidelines. This is most apparent in the lack of funding for later lines of therapy in most of Canada, despite guidelines recommending continued anti-HER2 therapy (Table 2).

The National Comprehensive Cancer Network (NCCN) [35] and European Society for Medical Oncology (ESMO) [36] guidelines for the treatment of metastatic or unresectable HER2-positive breast cancer recommend pertuzumab + trastuzumab and a taxane as first-line therapy (Table A1). For second-line therapy, the NCCN and ESMO guidelines now both recommend T-DXd instead of T-DM1 as the preferred therapy, based upon the DESTINY Breast-03 trial, presented in 2021 [25,26]. With the arrival of new treatment options and clinical trials examining the efficacy of these agents in patients with brain metastases [28,37], the ESMO guidelines now further stratify treatment recommendations by patients with/without active brain metastases, with tucatinib + capecitabine + trastuzumab preferred over T-DXd for patients with active, untreated brain metastases.

Critical data on the efficacy of novel anti-HER2-positive therapies in the metastatic setting have emerged, highlighting the need for evolving treatment algorithms in this setting. The current paper reviews the latest data on anti-HER2 therapies in patients with metastatic or unresectable HER2-positive breast cancer. It discusses how these data may influence future clinical practice in Canada, focusing on second and third-line treatment, given the recent emergence of practice-changing studies in this setting. 

## 2. Treatment in the First-Line Setting

The CLEOPATRA trial examined the addition of pertuzumab to the doublet trastuzumab + docetaxel as first-line treatment in patients with HER2-positive metastatic breast cancer [38]. Results demonstrated an improvement in median progression-free survival (PFS; 18.5 vs. 12.4 months; HR 0.62; *p* < 0.001) and an even greater improvement in overall survival (OS; 57.1 vs. 40.8 months; HR 0.69; 95% CI 0.58–0.82 months) with the addition of pertuzumab to the doublet in the first-line setting [39]. As a result of the CLEOPATRA trial, pertuzumab + trastuzumab + docetaxel was approved as the standard of care for the treatment of patients with HER2-positive metastatic breast cancer who have not received prior anti-HER2 therapy or chemotherapy for metastatic disease [11]. 

In a more recent analysis after a median follow-up of 99.9 months, the 8-year landmark OS rates were 37% (95% CI 31–42) with the addition of pertuzumab and 23% (19–28) in the doublet group, demonstrating a long-term survival benefit after first-line treatment with the addition of pertuzumab [38]. Moreover, the 8-year landmark PFS rates were 16% (304 events) with the addition of pertuzumab and 10% (324 events) in the doublet group, suggesting that a subgroup of patients might be cured after first-line treatment. Patients who present with de novo metastatic disease, not previously exposed to HER2-targeting agents, are more likely to experience long-term disease control, particularly with oligometastatic disease [40]. PFS and OS estimates are strikingly good for these patients if they can achieve a no-evidence-of-disease (NED) status.

## 3. Treatment for Relapse Following First-Line Therapy

Following first-line therapy, a number of treatment options have been examined over the years in the relapsed setting, including ADCs, small-molecule TKIs, and combinations of these agents or chemotherapies with MAbs [35,36]. A summary of phase II/III trials in chronological order is presented in Table 2.

Lapatinib was the first orally active small molecule that inhibits the intracellular tyrosine kinase domains of HER2 and EGFR [20]. A phase III trial published in 2006 in metastatic HER2-positive breast cancer patients who progressed after regimens that included, but were not limited to, anthracyclines, taxanes, and trastuzumab, compared lapatinib + capecitabine to capecitabine monotherapy. Results demonstrated an improvement in time to progression (median 8.4 vs. median 4.4 months; *p* < 0.001) with the addition of lapatinib (Table 2). However, there was no statistically significant difference in median OS between treatment groups (*p* = 0.72). Lapatinib was then approved by Health Canada in 2013 in the third-line metastatic setting in combination with capecitabine [13,41].

Trastuzumab emtansine (T-DM1) is an ADC that includes the HER2-targeted antitumor properties of trastuzumab, covalently linked to the cytotoxic microtubule inhibitor, DM1 [21]. In the phase III EMILIA trial published in 2012, the efficacy of T-DM1 was compared to that of lapatinib + capecitabine in the second-line setting in patients with HER2-positive advanced breast cancer who had previously been treated with trastuzumab and a taxane [21]. Results demonstrated superior PFS (median 9.6 vs. 6.4 months; *p* < 0.001) and OS (median 30.9 vs. 25.1 months; HR 0.68; *p* < 0.001) with T-DM1 versus lapatinib + capecitabine, with an improved safety profile. Likewise, the phase III TH3RESA trial published in 2014 also demonstrated an improvement in PFS (median 6.2 vs. 3.3 months; *p* < 0.0001) and OS (median 22.7 vs. 15.8 months; *p* = 0.0007) with T-DM1 versus physician’s choice in patients with advanced HER2-positive breast cancer who had received two or more HER2-directed regimens in the advanced setting, including trastuzumab and lapatinib, and previous taxane therapy [22,23]. T-DM1 was approved by Health Canada in 2013 in the metastatic setting in patients who have received trastuzumab and a taxane, separately or in combination [15,42].

Neratinib is an irreversible pan-HER TKI that has been examined in the phase III NALA trial published in 2020 in combination with capecitabine and compared to lapatinib + capecitabine in patients with HER2-positive metastatic breast cancer patients in the third-line setting, with two or more previous HER2-directed regimens [24]. Results of the study showed an improvement in mean PFS (8.8 vs. 6.6 months; HR 0.76; *p* = 0.0059) and reduced cumulative incidence of interventions for central nervous system (CNS) disease (22.8% vs. 29.2%; *p* = 0.043) with neratinib + capecitabine versus lapatinib + capecitabine. However, there was no significant improvement in mean OS with neratinib + capecitabine. Neratinib was approved by Health Canada in 2021 in combination with capecitabine in patients who have received two or more prior anti-HER2 regimens in the metastatic setting [14,43].

Trastuzumab-deruxtecan (T-DXd) is an ADC composed of an anti-HER2 antibody, a cleavable tetrapeptide-based linker, and a cytotoxic topoisomerase I inhibitor [32]. In the phase II DESTINY Breast01 trial published in 2019, after a median duration of follow-up of 11.1 months, T-DXd demonstrated an ORR of 60.9% in heavily pre-treated (median 6 previous regimens) patients with HER2-positive breast cancer [32]. An updated analysis reported a median PFS of 19.4 months after a median follow-up of 26.5 months [34]. Subsequently, T-DXd was examined in the phase III DESTINY Breast03 trial and compared to T-DM1 in patients with HER2-positive breast cancer in the second-line setting who were previously treated with trastuzumab and a taxane [25]. Interim results after a median follow-up of 16.2 months in the T-DXd group demonstrated an improved median PFS (T-DXd: NR vs. T-DM1: 6.8 months; *p* = 7.8 × 10^−22^) and an immature OS rate at 12 months showing a trend for benefit with T-DXd versus T-DM1 (94.1% (95% CI, 90.3–96.4) vs. 85.9% (95% CI, 80.9–89.7)). While patients with active brain metastases were not allowed in the trial, in a subgroup analysis in 82 patients with stable brain metastases, T-DXd reduced the risk of disease progression or death by 75% relative to T-DM1 [26]. The median PFS in this subgroup was 15.0 months with T-DXd and 3.0 months with T-DM1 (HR, 0.25; 95% CI, 0.13–0.45). T-DXd was approved by Health Canada in 2021 and is currently indicated in patients with unresectable or metastatic HER2-positive breast cancer who received prior treatment with T-DM1 [16,44]. However, new CADTH and INESSS applications were recently submitted for approval in the second line metastatic setting, including after recurrence during or within 6 months of completing neoadjuvant or adjuvant therapy. 

Tucatinib is an orally bioavailable, potent small-molecule TKI that is highly selective for HER2, without significant inhibition of EGFR [28]. In the large phase II randomized comparative HER2CLIMB trial published in 2019, the addition of tucatinib to trastuzumab + capecitabine resulted in an improvement in median PFS (7.8 vs. 5.6 months; *p* < 0.001) and median OS (21.9 vs. 17.4 months; *p* = 0.005) in patients with HER2-positive breast cancer who had received prior trastuzumab, pertuzumab, and T-DM1. After a median follow-up of 29.6 months, subsequent analysis showed the significant benefit in PFS and OS was maintained in all patients, including those with and without brain metastases at baseline [31]. In fact, HER2CLIMB was the first large trial that did not exclude patients with active brain metastases [37]. In an exploratory analysis of HER2CLIMB in 291 patients with brain metastases at baseline, the addition of tucatinib to trastuzumab and capecitabine doubled the intracranial ORR (47.3% vs. 20.0%; *p* = 0.03), reduced the risk of intracranial progression or death by two thirds (HR 0.32; *p* < 0.0001), and reduced the risk of death by nearly half (HR 0.58; *p* = 0.005). A subsequent analysis after 29.6 months of follow-up showed a 9.6-month improvement in median OS in patients with active brain metastasis (HR 0.524; *p* = 0.00087) and a 5.2-month improvement in patients with treated stable brain metastasis (HR 0.695; *p* = 0.1622) with the addition of tucatinib [30]. This study was the first to demonstrate that systemic therapy could effectively control brain metastasis while showing a meaningful improvement in OS. It established the combination of tucatinib + trastuzumab + capecitabine as the preferred treatment choice in this population. Health Canada approved Tucatinib in 2020 in combination with capecitabine and trastuzumab in patients who have received prior trastuzumab, pertuzumab, and T-DM1, separately or in combination [18,45].

Margetuximab is a chimeric, Fc-engineered, immune-activating anti-HER2 immunoglobulin G1 (IgG1) MAb that shares epitope specificity and Fc-independent antiproliferative effects with trastuzumab [27]. In the phase III SOPHIA trial published in 2021, margetuximab was compared to trastuzumab, each combined with chemotherapy, in patients with metastatic HER2-positive breast cancer. More than 30% of patients received greater or equal to two previous lines of therapy for advanced HER2-positive disease. Margetuximab improved median PFS over trastuzumab (5.8 (95% CI, 5.5–7.0) months vs. 4.9 (95% CI, 4.2–5.6) months; *p* = 0.03). However, there was no statistically significant improvement in median OS. Margetuximab is not currently approved by Health Canada. 

In addition to the above therapies, trastuzumab duocarmazine and pyrotinib are two anti-HER2 therapies with immature follow-up thus far, yet still reporting promising efficacy data, which are worth noting. Trastuzumab duocarmazine (SYD985) is a HER2-targeting ADC that includes trastuzumab bound to a linker drug-containing duocarmycin [46]. In the phase 3 TULIP trial, 437 patients with a median of 4 prior lines of therapy were randomized to receive trastuzumab duocarmazine or the physician’s choice of treatment. A preliminary analysis showed a median PFS for trastuzumab duocarmazine of 7.0 months (95% CI 5.4–7.2) versus 4.9 months (4.0–5.5) in the physician’s choice arm (HR 0.64; *p* = 0.002). There was no significant difference in OS between groups. Pyrotinib is a novel irreversible epidermal growth factor receptor/HER2 dual TKI [47]. In the phase 3 PHOEBE trial, 267 Asian patients from China were randomized to receive pyrotinib or lapatinib plus capecitabine. In an interim analysis, median PFS was significantly longer with pyrotinib plus capecitabine (12.5 months) versus lapatinib plus capecitabine (6.8 months) (HR 0.39; *p* < 0.0001). In an updated analysis, with a median follow-up of 33.2 months in the pyrotinib plus capecitabine arm and 31.8 months in the lapatinib plus capecitabine arm, median OS was not reached for pyrotinib + capecitabine versus 26.9 months for lapatinib + capecitabine (HR 0.69; *p* = 0.02) [48]. Longer follow-up and additional studies should aid in clarifying the potential role of these regimens in the treatment of HER2-positive breast cancer. At this point, neither trastuzumab duocarmazine nor pyrotinib has been approved by Health Canada.

## 4. Canadian Perspective and Recommendations

**Figure 2:**
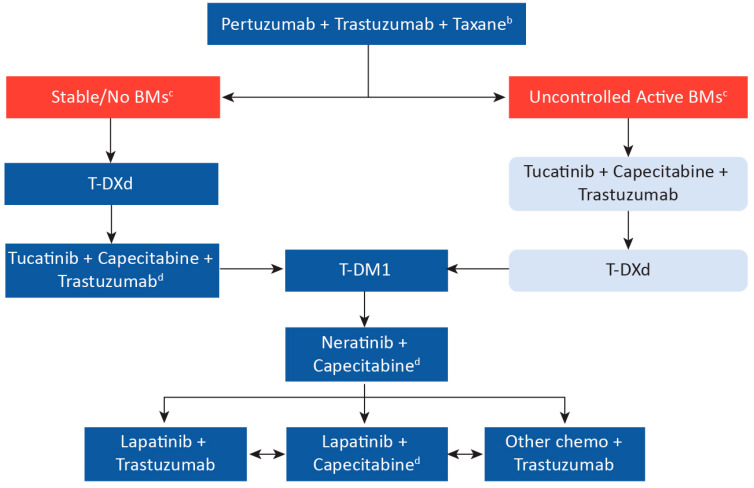
Suggested Treatment Sequence Algorithm for Metastatic HER2-Positive Breast Cancer in Canada Based on Clinical Trial Data ^a^. ^a^ Arrows represent treatment sequencing upon disease progression or toxicity and are suggestions only; clinical judgment must always be considered. Note that clinical data is based on historical treatment sequencing, and there remain data gaps in sequencing (e.g., use of T-DM1 following T-DXd). However, despite gaps in data, continual suppression of HER2 is considered critical. ^b^ Consider the response length if pertuzumab was used in the neoadjuvant setting. ^c^ Prior review with radiation oncology should be completed. As per the ASCO 2018 guidelines, patients with symptoms or a history of brain metastases should undergo imaging for brain metastases. However, we would consider baseline brain imaging also for untreated asymptomatic patients. Where local intervention with radiotherapy and/or surgery is not indicated, patients with uncontrolled BMs, or in the minority of patients with BMs and no visceral disease, we recommend tucatinib + capecitabine + trastuzumab. All patients should also be considered for clinical trials. ^d^ If no previous exposure to capecitabine. BM, brain metastases; T-DM1, ado-trastuzumab emtansine; T-DXd, trastuzumab deruxtecan.

Based on the clinical trial evidence to date and considering the Canadian landscape, we propose a practical treatment sequencing algorithm (Figure 2) for metastatic HER2-positive breast cancer in Canada. These recommendations should be seen as suggestions only, and clinical judgment should always be considered. Moreover, it is noted that any relevant Canadian clinical trials should always be considered as an option for all patients with metastatic HER2-positive breast cancer.

With improvement to systemic therapies for patients with HER2-positive breast cancer resulting in longer survival, the incidence of brain metastases has increased, developing in up to half of the patients during the course of their disease [49,50,51,52]. Moreover, patients with HER2-positive breast cancer are close to three times as likely to be diagnosed with brain metastases [53]. However, the role of brain imaging in a patient with recurrent disease is controversial, and there is no consensus. According to the ASCO 2018 guidelines for the treatment of patients with HER2-positive metastatic breast cancer, clinicians should not perform routine surveillance for brain metastases without a previous history or indicative symptoms [54]. However, these recommendations are now four years old and precede the newer evidence from HER2CLIMB, where approximately 50% of all HER2-positive metastatic breast cancer patients had brain metastases at baseline, based on mandated brain magnetic resonance imaging (MRI) screening, with 23% being untreated (unknown) [28,37]. The most recent ESMO guidelines suggest that brain imaging in patients with asymptomatic metastatic HER2-positive breast cancer may be justified if it will alter the treatment course [36]. As the finding of CNS disease may impact both local and systemic therapy, the authors of this manuscript recommend brain imaging at the time of the diagnosis of advanced disease or disease progression to have more comprehensive staging and help tailor treatment. In addition, because of the poor prognosis of patients with brain metastases, it is important to monitor for symptoms throughout the disease course.

## 5. First-Line Treatment

Based on the results of the CLEOPATRA trial, we recommend that pertuzumab + trastuzumab + a taxane remain the standard of care for the treatment of patients with HER2-positive metastatic breast cancer who have not received prior anti-HER2 therapy or chemotherapy for metastatic disease [11]. However, if patients were previously exposed to pertuzumab in the neoadjuvant/adjuvant setting, it is important to consider the disease-free interval (DFI) when deciding whether to repeat treatment with pertuzumab with evidence of recurrent disease. A DFI of less than 6 months may not warrant re-treatment with trastuzumab + pertuzumab, and the subsequent line of treatment can then be considered. First-line treatment for patients with brain metastases will remain pertuzumab + trastuzumab + a taxane, given the lack of studies examining this patient population in the first-line setting.

## 6. Second-Line Treatment

T-DM1 was considered the standard of care in the second-line setting in Canada based on the EMILA study [21]. However, T-DM1 is also indicated in the adjuvant setting in Canada for patients who have residual invasive disease following neoadjuvant taxane and trastuzumab-based treatment [19,55]. Given the results of the DESTINY-Breast03 trial, which reported a significant, large improvement in median PFS with T-DXd over T-DM1, we now recommend T-DXd as the preferred treatment option in the second-line setting. 

### Patients with Brain Metastases

Penetration of antibody-based anti-HER2 agents, such as trastuzumab, pertuzumab, and ADCs across an intact blood-brain barrier is thought to be limited [56]. However, tucatinib and its metabolites have been shown to effectively distribute to the cerebrospinal fluid [57]. It is of note that HER2CLIMB demonstrated effective treatment with only systemic therapy alone, including tucatinib + trastuzumab + capecitabine, rather than standard local therapies, such as neurosurgical resection and stereotactic (or whole-brain) radiation therapy [28]. This is an area of active research, with novel therapeutics leading to CNS penetrance and effective therapy for brain metastases. As a result of the improvement in outcomes for patients with brain metastases in the HER2CLIMB trial [28,37], we recommend that tucatinib + trastuzumab + capecitabine be favored as second-line treatment in patients with metastatic HER2-positive breast cancer with uncontrolled active brain metastases.

## 7. Third-Line Treatment

Third-line treatment of metastatic HER2-positive breast cancer and beyond is complicated by a lack of studies examining the efficacy of treatment following novel therapies such as T-DXd, as they were not used in earlier lines of therapy at the time of historical clinical trials. However, the importance of continual HER2 blockade has been established in clinical trials demonstrating the benefit of using trastuzumab beyond progression [58,59]. We believe that, when available, all lines of therapy for metastatic HER2-positive breast cancer should include anti-HER2 agents to maintain ongoing suppression of the HER2 pathway signaling. The availability of five new trastuzumab biosimilars may allow for potential ongoing HER2 blockade in these later line settings [60].

The demonstrated OS improvement with the addition of tucatinib to trastuzumab + capecitabine was documented in the HER2CLIMB trial, which included patients previously exposed to T-DM1 [28,37]. We believe it reasonable to extrapolate from these results to suggest that this combination should be the preferred option in the third-line setting in patients not previously exposed to capecitabine, and previously exposed to an ADC, whether T-DM1 or T-DXd (Figure 2). In addition, results from the EMILIA [21] and TH3RESA [22,23] trials suggest that T-DM1 also remains a reasonable option in the third-line setting. However, in patients progressing after T-DXd, the triplet tucatinib + capecitabine + trastuzumab may be preferable to T-DM1, prioritizing a regimen with a different mechanism of action while still leaving T-DM1 for later lines of therapy.

### Patients with Brain Metastases

T-DXd is a reasonable third-line option in metastatic patients with active brain metastases who received tucatinib + capecitabine + trastuzumab as second-line therapy.

## 8. Subsequent Treatments

Based on the results of the TH3RESA trial in metastatic patients with two or more previous lines of therapy, T-DM1 is a reasonable option in this setting [22] (Figure 2). Neratinib is preferred over lapatinib in combination with capecitabine based on a demonstrated PFS benefit in the NALA trial, which included metastatic patients with two or more previous lines of therapy [24]. However, other options may be preferable in patients previously exposed to capecitabine. Margetuximab also demonstrated an improvement in PFS over trastuzumab in the SOPHIA trial [27]. As margetuximab is not available in Canada, for patients previously exposed to capecitabine, we would recommend either novel therapies available through clinical trials or continuous trastuzumab combined with other chemotherapies (such as vinorelbine) [61].

## 9. Summary

In recent years, the development of a number of effective anti-HER2 targeted treatments has revolutionized the treatment landscape in HER2-positive metastatic breast cancer. In the first-line setting, pertuzumab + trastuzumab + taxane provides a durable benefit in a significant proportion of patients. In the second-line setting, clinical trials show T-DXd is a highly effective option, resulting in a shift from T-DM1 as the previous standard of care. Moreover, we now have data in a subgroup of these patients with brain metastases to show that tucatinib + trastuzumab + capecitabine can improve survival in this higher-risk group and be an effective regimen for all patients in the third-line setting. In addition, we have a number of effective anti-HER2 therapies that can be used in subsequent lines of therapy to improve patient outcomes.

In the next three to five years, we expect to see continued improvements in outcomes with the development of novel anti-HER2 agents, such as the bispecific antibody ZW25 (zanidatamab) and its corresponding ADC, ZW49 (zanidatamab linked with a cytotoxin), as well as the novel ADC, ARX788 (ACE-Breast03; NCT04829604) [62,63]. There are also ongoing trials combining HER2-targeting agents with atezolizumab immunotherapy either in all comers (e.g., NCT03199885) or in patients selected for PD-L1 positive disease (e.g., KATE3; NCT04740918). We are also making strides in our understanding of various HER2-positive subtypes, such as estrogen receptor-positive patients, who may benefit from other types of therapy. For example, results of the monarcHER study demonstrated promising efficacy in this subgroup with abemaciclib, a potent oral cyclin-dependent kinase 4 (CDK4) and 6 (CDK6) inhibitor, combined with trastuzumab + fulvestrant to produce an effective chemotherapy-free regimen [64].

Given the rapid change to the treatment landscape for HER2-positive breast cancer, it has become a priority to accelerate drug approval and access to therapies for patients in Canada. However, it is also critical that clinical trials be designed to produce meaningful outcomes, allowing for more rapid worldwide access. We hope that as newer data emerges, Canadians can access these promising novel agents to provide the best outcomes for patients with metastatic HER2-positive breast cancer.

## Figures and Tables

**Table 1:** Availability of Anti-HER2 Therapies for Metastatic Breast Cancer in Canada as of March 2022.

Agent	Health Canada Indication	Recommendations for Funding in Canada
Trastuzumab [10]	In patients with HER2-positive metastatic breast cancer	CADTH: None INESSS: Positive
Pertuzumab [11]	In combination with trastuzumab + docetaxel with no prior anti-HER2 therapy or chemotherapy for metastatic disease	CADTH: Reimburse with clinical criteria and/or conditions INESSS: Positive
Tucatinib [18]	In combination with trastuzumab + capecitabine where have received prior trastuzumab, pertuzumab, and trastuzumab emtansine, separately or in combination	CADTH: Reimburse with clinical criteria and/or conditions INESSS: Positive
Lapatinib [13]	In combination with capecitabine where patients progressed on taxanes and anthracycline before starting therapy Patients should have progressed on prior trastuzumab therapy in the metastatic setting	CADTH: Do not reimburse INESSS: Positive
Neratinib [14]	In combination with capecitabine in patients who have received two or more prior anti-HER2-based regimens in the metastatic setting	CADTH: Do not reimburse INESSS: Not submitted
T-DM1 [19]	In patients who received both trastuzumab and a taxane, separately or in combination Patients should have either received prior therapy for metastatic disease, or developed disease recurrence during or within 6 months of completing adjuvant therapy	CADTH: Reimburse with clinical criteria and/or conditions INESSS: Positive
T-DXd [16]	In patients who have received prior treatment with T-DM1	CADTH: Submitted for new second-line indication INESSS: Submitted for new second-line indication

T-DM1, ado-trastuzumab emtansine; T-DXd, trastuzumab deruxtecan.

**Table 2:** Phase II/III Clinical Trials on the Efficacy of Anti-HER2 Therapies in the Relapsed Setting.

Study/N	Treatment Arms	Prior Treatment	Primary Outcome	Select Secondary Outcomes
Phase III Clinical Trials				
Geyer et al. [20] (N = 399)	Lapatinib + capecitabine vs. capecitabine	Anthracyclines, taxanes, and trastuzumab	Time to progression: 8.4 vs. 4.4 mos (HR 0.49; *p* < 0.001)	ORR: 22% vs. 14% (*p* = 0.09) No OS difference (*p* = 0.72)
EMILIA [21] (N = 991)	T-DM1 vs. Lapatinib + capecitabine	Trastuzumab + taxane	Median PFS: 9.6 vs. 6.4 mos (HR 0.65; *p* <0.001) Median OS: 30.9 vs. 25.1 mos (HR 0.68; *p* < 0.001)	ORR: 43.6% vs. 30.8% (*p* < 0.001)
TH3RESA [22,23] (N = 602)	T-DM1 vs. physician’s choice	Taxane, lapatinib, and ≥2 HER2-targeted regimens, including trastuzumab	Median PFS: 6.2 vs. 3.3 mos (HR 0.53; *p* < 0.0001) Median OS: 22.7 vs. 15.8 mos (HR 0.68; *p* = 0.0007)	ORR: 3.31 vs. 8.6% (*p* < 0.0001)
NALA [24] (N = 621)	Neratinib + capecitabine vs. lapatinib + capecitabine	Trastuzumab, pertuzumab, or T-DM1	Mean PFS: 8.8 vs. 6.6 mos (HR 0.76; *p* = 0.0059) Mean OS: 24.0 vs. 22.2 mos (HR 0.88; *p* = 0.21)	ORR: 32.8 vs. 26.7% (*p* = 0.12) Cumulative incidence of interventions for CNS disease: 22.8% vs. 29.2% (*p* = 0.043)
DESTINY-Breast03 [25,26] (N = 524)	T-DXd vs. T-DM1	Trastuzumab (99.6%), pertuzumab (61.1%), other anti-HER2 (15.9%) 1 prior therapy: 48.3%	Median PFS: T-DXd: NR (18.5-NE) vs. T-DM1: 6.8 mos (5.6–8.2) (HR 0.28, *p* = 7.8 × 10^−22^)	Est. OS at 12 mos: T-DXd: 94.1% (95% CI, 90.3–96.4) vs. T-DM1: 85.9% (95% CI, 80.9–89.7) Median PFS (INV): T-DXd: 25.1 (22.1-NE) vs. T-DM1: 7.2 (6.8–8.3) (HR 0.26, *p* = 6.5 × 10^−24^) Patients with BMs: Median PFS: T-DXd: 15.0 mos vs. T-DM1: 3.0 mos (HR, 0.25; 95% CI, 0.13–0.45)
SOPHIA [27] (N = 536)	Margetuximab vs. trastuzumab	Trastuzumab (100%), pertuzumab (100%), T-DM1 (91.0%)	Median PFS: 5.8 vs. 4.9 mos (HR 0.76; *p* = 0.03) Median OS: 21.6 vs. 19.8 mos (HR 0.89; *p* = 0.33)	24% relative risk reduction for PFS favoring margetuximab (HR 0.76 *p* = 0.03)
Phase II Clinical Trials				
HER2CLIMB [28,29,30,31] (N = 612)	Tucatinib + trastuzumab + capecitabine vs. trastuzumab + capecitabine	Trastuzumab, pertuzumab, and T-DM1	14-month follow-up: Median PFS: 7.8 vs. 5.6 mos (HR 0.54; *p* < 0.001) 29.6-month follow-up: Median PFS: 7.6 vs. 4.9 mos (HR 0.57; *p* < 0.00001)	14-month follow-up: Median OS: 21.9 vs. 17.4 mos (HR 0.66; *p* = 0.005) ORR: 40.6 vs. 22.8% (*p* < 0.001) Median PFS in patients with BMs at baseline: 7.6 mos vs. 5.4 mos (HR 0.48; *p* < 0.001) 29.6-month follow-up: Median OS: 24.7 vs. 19.2 mos (HR 0.73; *p* = 0.004) Significant differences remained in those with/without visceral metastases Active BMs: Median OS: 21.4 vs. 11.8 mos (HR 0.524; *p* = 0.00087) Stable BMs: 21.6 vs. 16.4 mos (HR 0.695; *p* = 0.162)
DESTINY-Breast01 [32,33,34] (N = 184)	T-DXd	Trastuzumab (100%), T-DM1 (100%), pertuzumab, other HER2-targeted Tx, hormone therapy, other systemic Tx Median (range): 6 (2–27)	11.1-month follow-up: ORR: 60.9% (95% CI, 53.4–68.0) 26.5-month follow-up: 62.0% (95% CI, 54.5–69.0)	11.1-month follow-up: Median PFS: 16.4 mos (95% CI, 12.7–NR) Est. OS at 12 mos: 86.2% (95% CI, 79.8–90.7) 26.5-month follow-up: Median PFS: 19.4 mos (95% CI, 14.0–25.0) Median OS (31.1-month follow-up): 29.1 mos (95% CI, 24.6–36.1)

BMs, brain metastases; CI, confidence interval; CNS, central nervous system; HR, hazard ratio; mos, months; NR, not reached; ORR, overall response rate; OS, overall survival; PFS, progression-free survival; T-DM1, ado-trastuzumab emtansine; T-DXd, trastuzumab deruxtecan.

## Appendix A

**Table A1:** Guidelines for the Treatment of Metastatic/Unresectable HER2-Positive Breast Cancer.

	NCCN 2022 Guidelines [35]			ESMO 2021 Guidelines [36]
First-line	Pertuzumab + trastuzumab + docetaxel Pertuzumab + trastuzumab + paclitaxel	Hormone Receptor+ Docetaxel (or paclitaxel) + trastuzumab + pertuzumab, then trastuzumab + pertuzumab + endocrine therapy until progression Chemo contraindicated: Trastuzumab +/− pertuzumab + endocrine therapy		Hormone Receptor− Docetaxel (or paclitaxel) + trastuzumab + pertuzumab, then pertuzumab + trastuzumab until progression Chemo contraindicated: Trastuzumab + pertuzumab until progression
Second-line	T-DXd (preferred) T-DM1	Active BMs		No/Unknown/Stable BMs
		Local intervention indicated: Resection, SRT or WBRT, depending on the number of BMs & prognostic factors	Local intervention not indicated: Tucatinib + capecitabine + trastuzumab (preferred) T-DM1	T-DXd (preferred) T-DM1
Third-line and Beyond (Other recommended regimens)	Tucatinib + trastuzumab + capecitabine Trastuzumab + docetaxel or vinorelbine Trastuzumab + paclitaxel ± carboplatin Capecitabine + trastuzumab or lapatinib Trastuzumab + lapatinib (w/out cytotoxic therapy) Trastuzumab + other agents Neratinib + capecitabine Margetuximab-cmkb + chemo *	Active BMs		No/Unknown/Stable BMs
		Local intervention indicated: Resection, SRT or WBRT, depending on the number of BMs & prognostic factors	Local intervention not indicated: Tucatinib + capecitabine + trastuzumab Followed by: T-DXd Followed by: Lapatinib + Trastuzumab Trastuzumab + chemo Margetuximab + chemo Neratinib + chemo	Tucatinib + capecitabine + trastuzumab T-DXd T-DM1 Followed by: Lapatinib + Trastuzumab Trastuzumab + chemo Margetuximab + chemo Neratinib + chemo

BM, brain metastases; ESMO, European Society of Medical Oncology; SRT, stereotactic radiotherapy; T-DXd, trastuzumab deruxtecan; T-DM1, trastuzumab emtansine; NCCN, National Comprehensive Cancer Network; WBRT, whole-brain radiotherapy. * Currently not Health Canada approved.
